# Determination of the Risk Factors That Influence Occurrence Time of Traffic Accidents with Survival Analysis

**Published:** 2018-08

**Authors:** Burcu ORALHAN, Ziya Gökalp GÖKTOLGA

**Affiliations:** 1. Dept. of Business Administration, Faculty of Economics and Administrative Sciences, Nuh Naci Yazgan University, Kayseri, Turkey; 2. Dept. of Econometrics, Faculty of Economics and Administrative Sciences, Cumhuriyet University, Sivas, Turkey

**Keywords:** Traffic accidents, Survival analysis, Life tables, Cox regression analysis

## Abstract

**Background:**

This study aimed to determine risk factors that occurrence time of traffic accidents. Traffic accident occurrence time is defined as the time between a driver’s getting his/her license and having the first accident, involving death or injury between 2008–2012 and there were investigated.

**Methods:**

This study was conducted with the Cox Regression and life tables models included among survival analysis models. Data of all 11.671 traffic accidents in Kayseri in Turkey were analyzed for the 5-yr period.

**Results:**

The non-occurrence rate of traffic accidents involving injury is mostly affected by gender, age, education, number of vehicles involved in accident, road surface material, daylight, type of road, direction of road and time of the day. The non-occurrence rate of fatal traffic accident duration is mostly affected by gender, age, education, daylight and horizontal alignment. The rate of having an accident involving death or injury after getting driver’s license is 30.3% in the first 5 yr, it is 50.1% in the first 10 yr and 91.7% in 25 yr.

**Conclusion:**

As the non-occurrence time increases, occurrence of accidents in earlier years will decrease. In other words, the number of accidents in earlier years will be lower. This will cause a decrease in the number of accidents in total.

## Introduction

Motor vehicles provide many benefits in human transportation and freight shipment by offering necessary speed, comfort and cost options. However, they also have created materialistic and nonmaterialistic damages as a result of the increasing number of accidents because the number of people who get a driving license and motor vehicles has increased. According to WHO, 1.240.000 people every year die in the world at traffic accidents (1). The financial harm resulting from accidents is $518 annually. The traffic accidents occurring on the road take up 2.1% of the causes of deaths in the world. Overall, 50000 people pass away in Europe every year (2).

Traffic accidents are one of the most important economic and social problems that cause many serious results including thousands of injuries and deaths in Turkey as in the world (3,4). Totally, 5000 people die in Turkey every year because of accidents and hundreds of thousands of people get injured or become disabled. Traffic problems are among the most important economic and social problems that breed various serious results, hundred thousands of people get injured, disabled or die too. Turkey follows the world in terms of the ratio of fatal accidents to the population (0.05 per thousand), the rate of accidents involving injury is 2.32 per thousand (5). Therefore, many studies in different countries are conducted with different analysis methods to decrease the number of accidents that cause serious damages. Latent Class Clustering and Bayesian Networks (6), Artificial neural networks (7), Binary logit and binary probit (8–11), Clustering Analysis (12), Multinomial logit (13–15), Nested logit (8,16), Kernel Density Estimation (17) and many different methodological approaches have been used to analyse on traffic accidents and severity (18,19). In traffic accident analysis is included many variables as injury severity, gender, age, seat belt, cause of crash, vehicle type, location type, lighting condition, weather condition, road surface, occurrence, speed, traffic volume, age of vehicle, alcohol, weight of vehicle, type of driving license (20–23).

Lapse between the times that a driver receives his/her license and has a traffic accident is not underlined enough. However, this data is one of the important variables in analysis of traffic accidents because delaying the time of an accident means a driver will have the accident much later. Survival analysis is used to analyze the effects of the variables that can influence time that passes through the endpoint. Survival analysis methods are not applied sufficiently in traffic accident studies. They are done in three groups of vehicles, pedestrians, and environmental factors. Pedestrian studies with survival analysis are in the USA (24) and in India (25). Environment factors as red light violations are investigated in 2011 (26). Another study is about the environment and vehicle risk factors of accident in winter (27). Bikers accidents were examined in 6 variables that influence traffic conditions (28). Vehicle accidents occurred were examined and investigated accidents involving death depending on vehicle types and models (29). Another study have been analyzed for accidents which have occured in China (30). Influence of collides in accidents have been emphasized in emergency health centers (31).

In this study, risk factor affecting the accidents which occurred in Kayseri in 5 yr were examined with Cox regression and life tables models that are among survival analysis models.

## Materials and Methods

**Fig. 1:**
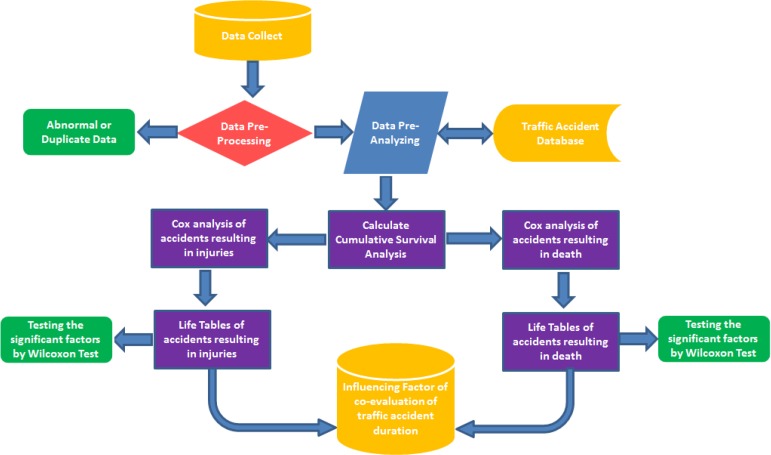
Process Flow

This study was a case-control study. Traffic accidents involving death or injury that occurred from 2008–2012 and their occurrence time were investigated. There were 79 accidents involving death and 11.592 involving injuries examined through accident reports. There were many variables that influence traffic accidents occurrence time as gender, age, education level, number of vehicles involved, location, road surface material, road type, daylight and etc. The dependent variable was accident occurrence time defined as the time between a driver’s getting his/her license and having the first accident. All variables were analyzed in the Cox regression and life tables model is believed to have an effect the dependent variable, defined as accident occurred time, distinguished as ‘dead’ or ‘insured’. (Fig. 1)

Survival analysis was used to analyze the effects of the variables that can influence time that passes till the accident. This model was based on the beginning and ending points. Firstly, endpoint of the study needs to be well defined. It is important to exclude the monitoring resulting from different incidents during the study from the observations. Another aspect was the state of non-occurrence of the expected incident at the end of the study and only knowing the beginning of this monitoring (32,33). Last aspect was irregularities in the dispersion of data. The variant analysis of such data using statistical methods because of stated reasons can be deceptive (34).

### Life Tables and Cox Regression Model

T is used to express lifespan (35). The cumulative distribution function (cdf), F(t) gives the likelihood of a variable to be lower than or equal to a pre-determined t-value.

S(t)=1−F(t)=P(T>t)

Hazard function is expressed as λ(t) and it shows failure rate in the state of T=t. Condition definite lifetime, t, is defined by taking its limit according to δ=Δt.

$$\lambda (t)=\underset{\delta \to 0}{\text{lim}}\frac{P(t\leq T<t+\delta |T\geq t)}{\delta }=\frac{P(t\leq T<t+\delta )}{P(T\geq t)\times \delta }=\frac{S(t)-S(t+\delta )}{\delta }\times \frac{1}{S(t)}=\frac{f(t)}{S(t)}$$

Cumulative hazard function is between [0, ∞] (36). When T lifespan is a defined continuous function, (*t*, *t* + Δ*t*) the likelihood of a monitoring to be failure at a determined time is T’s likelihood density function (37).

$$f(t)=\underset{\Delta t\to 0}{\text{lim}}\frac{P(t\leq T\leq t+\Delta t)}{\Delta t}$$

And the function is below (38).

$$\begin{array}{ccc} F(t)=P(T\leq t)=\int_{0}^{t}f(x)\mathrm{dx} & 0\leq t<\infty & f(t)=\frac{\mathrm{dF}(t)}{\mathrm{dt}} \end{array}$$

Life tables function is below.

$$S(t)=\prod_{j=1}^{k}\frac{n_{j}^{\prime }-d_{j}}{n_{j}^{\prime }}$$

Comparison of lifespan: It is used in the analyses that aim to compare lifespans of two groups that are different in lifespan distribution. As this data includes censored information, distribution of survival functions should be compared with special tests (39). In the detection of differences in monitoring groups censored from right, log-rank statistics and Wilcoxon test statistics are very powerful. For this reason, they are the most commonly used test statistics.

### Cox Regression Model

Regression analyses are the mathematical models showing the effectiveness levels of independent variables that are thought to explain the dependent variable. The dependent variable of lifespan examined in studies is the observation of the time that passes from an undetermined time to when the accident occurs. Independent variables are the ones that influence this time.

Let the independent variables that might be effective on lifespan be called as X. Total of independent variables is X= (X_1_, .....X_n_). In this case, if the hazard function *λ*(*t*), which is non-negative and has a baseline boundary hazard function *λ*_0_(*t*) is accepted,

$$\lambda (t)=\lambda_{0}(t)e^{f(x)}$$

In the Cox Regression Model, as we take the number of failure times as n, β coefficient is predicted with partial likelihood function L(β). Since the risk cannot be explained with parametric models’ likelihood inference cannot be applied to Cox Regression model. In this case, as it is not censored from left, β coefficients can be predicted by maximizing the partial likelihood function (40). This for β coefficient is defined. In the equation (41),

$$L(\beta )=\prod_{t=1}^{n}\prod_{t\geq 0}{\left\{\frac{Y_{i}(t)\text{exp}(\beta \prime X_{i})}{\sum_{j\in R_{n}}Y_{i}(t)\text{exp}(\beta \prime X_{i})}\right\}}^{dN_{i}(t)}$$

Yi (t): Inspected i individual’s risk at t time (1: there is risk, 0:no risk)
dNI (t): At a certainly limited time interval ([t, t+Δt]) Increase observed at Ni
Rn: Unities under risk at a certain time

β coefficient can be predicted by solving with Newton-Raphson algorithm. To test the value of β coefficients, Wald Test, Similarity Rate (Likelihood, LR) Test and Score Test can be used.

The factors influencing duration of traffic accidents have been investigated with survival analysis tables and Cox regression model (Method: Enter), the comparisons have been calculated according to Wilcoxon (Gehan) test statistics. There was not efficient data on drivers’ faults and sites of accidents in those involving death, these variables were excluded from the study. *P*<0.05 value was regarded as statistically significant in the analysis.

## Results

**Fig. 2:**
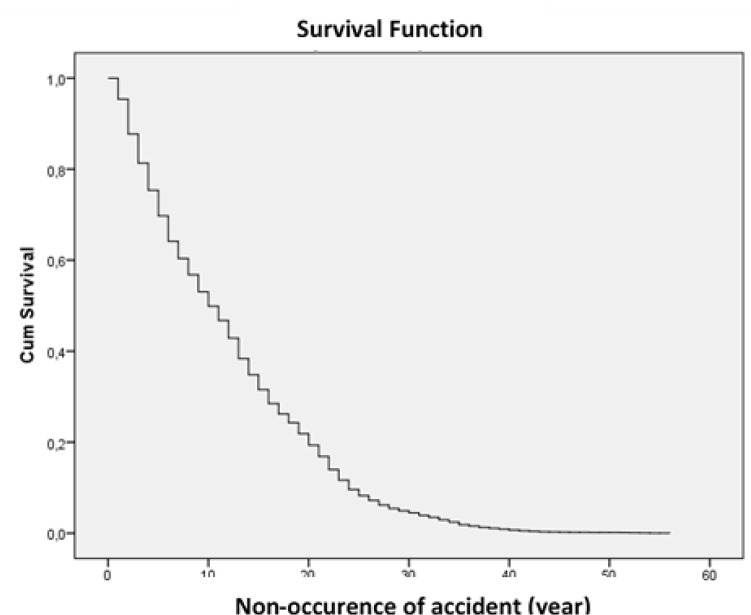
Non-occurence of accident rate survival function

From 2008 to 2012, 11671 people had a traffic accident from which 8607 (73.7%) were male and 3064 (26.2%) were female. Drivers 16.2% was 16–25, 41.9% was 26–35, 19.2% was 36–45, 10.7% was 46–55 and 12% was up to 55 age. Drivers education graduate level of 23.8% was primary, 15% was secondary, 39.3% was high, 21.8% was higher (Table 1). Non-occurrence of accident rate is given cumulatively in Table 2 and Fig. 2 below of the drivers involved in accidents during the period Jan 1st, 2008 and Dec 31st, 2012 after getting their driving licenses.

**Table 1:** Descriptive statistics of traffic accidents

***Variables***		***Accident Results***			
		***Fatal***		***Non-Fatal***	
		***n***	***%***	***n***	***%***
Weather conditions	Sunny	41	0.5	8973	99.5
	Rainy	38	1.4	2619	98.6
Day light	Daytime	27	0.3	8168	99.7
	Night	52	1.5	3424	98.5
Surface of road	Dry	45	0.5	9933	99.5
	Wet	34	2.0	1659	98.0
Road surface material	Concrete and other	2	4.8	40	95.2
	Asphalt	77	0.7	11552	99.3
Road Type	Divided road	41	0.5	8468	99.5
	Undivided road	38	1.2	3124	98.8
Road direction	Single direction road	34	0.4	8325	99.6
	Double direction road	45	1.4	3267	98.6
Horizontal alignment	Straight and level road	50	0.5	10823	99.5
	Curve	29	3.6	769	96.4
Vertical alignment	Level	53	0.5	10180	99.5
	Inclined	26	1.8	1412	98.2
Intersections	Not	3	1.2	247	98.8
	Available	76	0.7	11345	99.3
Passages	Not	67	0.6	10746	99.4
	Available	12	1.4	846	98.6

**Table 2:** Non-occurrence of accident rate

***Time Range***	***Non-occurrence of accident rate***	***Cumulative Non-occurrence of accident rate***	***Standard Error***
0–5	0.697	0.697	0.005
6–10	0.716	0.499	0.005
11–15	0.632	0.315	0.005
16–20	0.613	0.193	0.004
21–25	0.427	0.083	0.003
26–30	0.542	0.045	0.002
31–35	0.419	0.019	0.001
36–40	0.376	0.007	0.001
41–45	0.309	0.002	0.000
46–50	0.714	0.002	0.000
51–55	0.133	0.000	0.000
56–	0.000	0.000	0.000

**Fig. 3:**
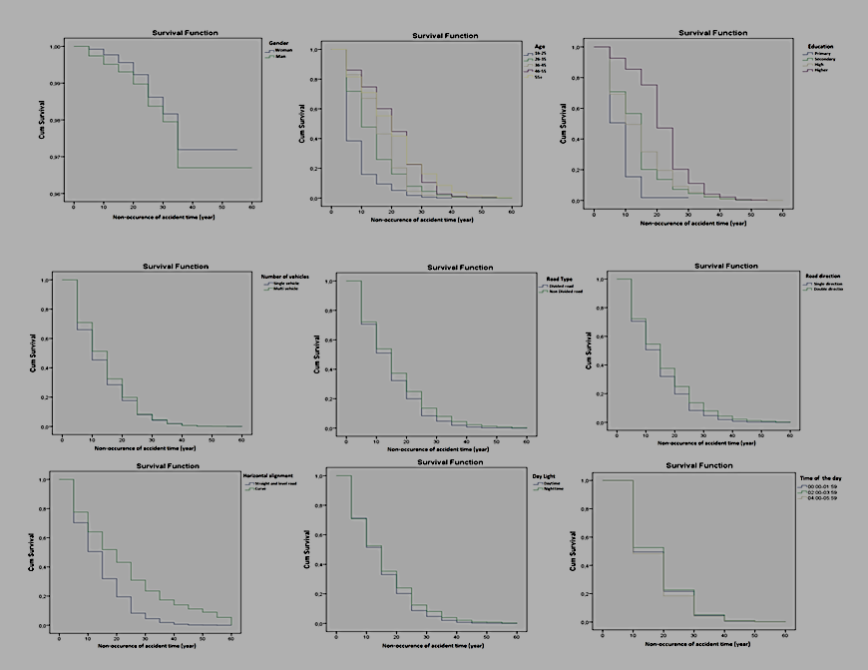
Results of survival functions of the variables of the accidents involving injury

The rate of having an accident involving death or injury after getting driver’s license is 30.3% in the first 5 yr, it is 50.1% in the first 10 yr and 91.7 in 25 yr. The effects of the determined factors on occurrence duration/time of the accidents are analyzed with 19 variables. According to the table, gender, education level, age, number of vehicles involving in the accident, daylight, covering type of the road, road type, directions and hours of the day are important factors. Cox regression analysis of non-fatal accidents results shown in Table 3 and Fig. 3.

**Table 3:** Cox regression analysis of non-fatal accidents results

***Variable***		***B***	***SE***	***Wald***	***Sd***	***p***	***HR***	***HR %95 GA***									
								***Min***	***Max***
Gender	Woman								
	Man	−0.24	0.023	105.093	1	<0.001	0.786	0.751	0.823
Education	Primary				3				
	Secondary	1.548	0.034	2042	1	<0.001	4.7	4.395	5.027
	High	0.726	0.033	471.865	1	<0.001	2.068	1.936	2.208
	Higher	0.586	0.027	474.787	1	<0.001	1.797	1.705	1.894
Age(yr)	16–25				4				
	26–35	1.125	0.04	790.742	1	<0.001	3.08	2.848	3.331
	36–45	0.48	0.034	204.238	1	<0.001	1.616	1.513	1.726
	46–55	0.422	0.038	121.409	1	<0.001	1.525	1.415	1.644
	>55	0.022	0.044	0.248	1	0.619	1.022	0.938	1.114
Number of vehicles involved in accident	Single vehicle								
	Multi vehicles	0.178	0.065	7.648	1	0.006	1.195	1.053	1.357
Day Light	Daytime								
	Night	0.09	0.035	6.388	1	0.011	1.094	1.02	1.172
Covering Type of the Road	Concrete and other Asphalt	−0.431	0.165	6.811	1	0.009	0.65	0.47	0.898
Road Type	Divided road								
	Undivided road	−0.117	0.05	5.536	1	0.019	0.889	0.806	0.981
Road direction	Single direction road								
	Double direction road	0.12	0.049	6.04	1	0.014	1.128	1.025	1.241
Time of the day	22:00–05:59				2				
	06:00–13:59	0.118	0.036	10.982	1	0.001	1.125	1.049	1.207
	14:00–21:59	−0.68	0.024	8.202	1	0.004	0.934	0.891	0.979

After getting the license, the duration of getting involved in an accident is 0.786 less among women than men. Compared to bachelor’s degree graduates, this risk is 4.7 times more among primary school graduates, 2.068 time more among secondary school graduates and 1.797 times more among high school graduates. In terms of age groups, compared to above 55 group, the risk is 3.080 times more among the group of 16–25; 1.616 times more among the group of 26–35; 1.525 times more among the group of 36–45. Accidents involving only one vehicle are 1.195 times riskier than the accidents involving two or more vehicles. The accidents occurred in daytime are 1.094 times riskier than those occurred at night. The accidents occurred on the roads covered with other substances other than asphalt are 0.650 times riskier than those occurred on the roads covered with asphalt.

**Fig. 4:**
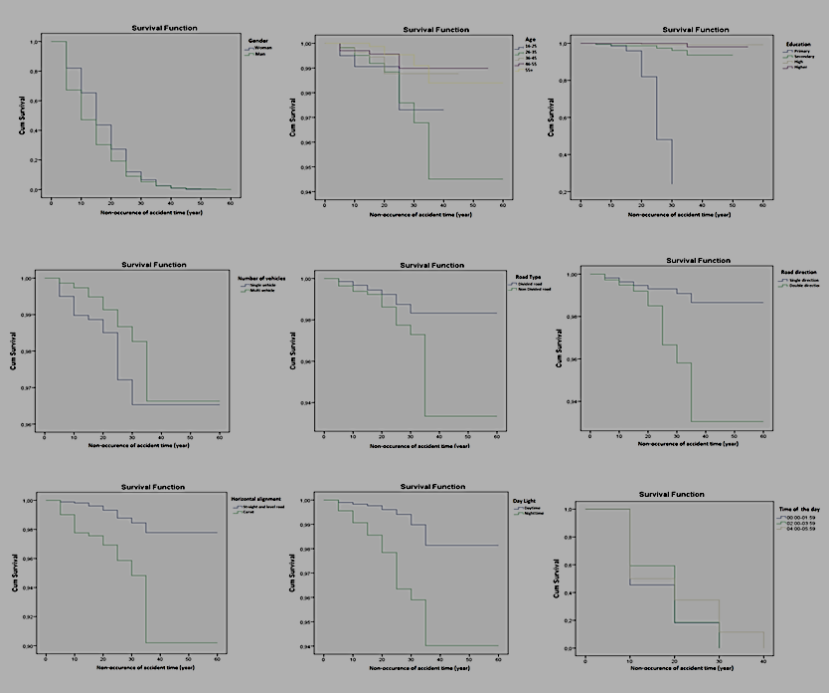
Results of survival functions of the variables of the accidents involving death

The accidents occurred on divided roads are 0.889 times riskier than those occurred on standard roads. The accidents occurred on one-sided roads are 1.128 times riskier than those on two-sided roads. There is a significant difference among the accidents occurred during the periods of 22:00–05:59, 06:00–13:59 and 14:00–21:59. Cox regression analysis of fatal accidents results shown in Table 4 and Fig. 4.

**Table 4:** Results of Cox regression analysis of fatal accidents

***Variable***		***B ***	***SE ***	***Wald ***	***Sd ***	***p ***	***HR ***	***HR %95 GA***									
								***Min ***	***Max ***
Gender	Woman								
	Man	−1.53	0.432	12.526	1	<0.001	0.217	0.093	0.505
Education	Primary				3				
	Secondary	3.993	0.615	42.181	1	<0.001	54.236	16.252	180.99
	High	3.192	0.581	30.223	1	<0.001	24.346	7.801	75.983
	Higher	0.803	0.619	1.685	1	0.194	2.233	0.664	7.507
Age(yr)	16–25				4				
	26–35	1.157	0.684	2.864	1	0.091	3.18	0.833	12.145
	36–45	1.074	0.576	3.469	1	0.063	2.926	0.945	9.056
	46–55	0.364	0.648	0.315	1	0.575	1.439	0.404	5.128
	>55	0.157	0.696	0.051	1	0.821	1.17	0.299	4.577
Day Light	Daytime								
	Night	−3.13	0.449	48.568	1	<0.001	0.044	0.018	0.105
Horizontal alignment	Straight and level road								
	Curve	−2.011	0.37	29.537	1	<0.001	0.134	0.065	0.276
Time of the day	22:00–05:59				2				
	06:00–13:59	−0.122	0.378	0.104	1	0.747	0.885	0.422	1.858
	14:00–21:59	1.539	0.398	14.925	1	<0.001	4.661	2.135	10.178

When the table is examined for the duration of occurrence of accident; variables such as gender, education level, daylight, horizontal alignment and time of the day can be stated as notable. The risk of having an accident involving death is 0.216 times less among women than men. It is 54.236 times more for primary school graduates, 24.346 times more for secondary school graduates than bachelor’s degree graduates. The risk of involving death in an accident is 0.044 times less in daytime than at night. The accidents on straight roads are 0.134 time riskier than those on slightly crooked roads. There is no significant difference among the periods of 22:00–05:59 and 16:00–13:59 while there is such a notable difference among the periods of 22:00–05:59 and 14:00–21:59.

According to Wilcoxon (Gehan) Statistic, there was a significant difference between the age groups of 46–55 and above 55 and the rest (*P*<0.05); between 16–25 and above 55; 26–35 and above 55 (*P*<0.05) and between the groups of 36–45 and above 55 (*P*<0.05). There was statistical difference among those groups’ involvement times in accidents. The nonoccurrence time of accidents increased from primary school to bachelor’s degree. According to Wilcoxon (Gehan) Statistics, there was no significant difference between high school graduates and secondary school graduates in having accidents involving injuries as it was the case between primary school graduates and secondary school graduates in accidents involving death. However, there was a serious difference for these groups in other cases. Accidents involving one vehicle happened earlier than accidents involving two or more vehicles, the duration till having accidents at night was shorter, the type of road did not affect the results of accidents. Having accidents involving death or injuries on divided roads was more common, as so on one-way-roads compared to two-ways-roads. Accidents involving death happened faster on slightly crooked roads; however, accidents involving injuries happen faster on straight roads. Accidents involving death happened faster between the hours 00:00–07:59, accidents involving injuries happened faster between the hours 15:59–00:00.

## Discussion

This study helps us to understand the effect of variables about reasons of traffic accidents and accident occurrence time which is defined as the time between a driver’s getting his/her license and having the first accident. Survival analysis has been used to find the correlation between nonoccurrence time and determined variables about reasons of traffic accidents. In literature lots of the papers are examined for the analysis of factors associated with traffic injury but none of these are not interested with accident occurrence time. Some papers have been studied the relationship between detection, response, clearance, recovery time, and effects of traffic accident variables etc. (31, 42–44).

In this study, it was aimed to research the risk factors that influence occurrence time of traffic accidents with survival analysis that happened in Kayseri, Turkey during 2008–2012. When the results were examined, the chance of having an accident involving death or personal injuries within the first 5 yr after getting the driving license is 30.3%, within the first 10 yr is 50.1% and within 25 yr is 91.7%.

Delaying the non-occurrence time had many benefits for the drivers, accidents, those involved in accidents, their relatives, the economy of the country and the people individually as well as the personal and societal psychology. These benefits are;

1. As the non-occurrence time increases, occurrence of accidents at earlier years will decrease. In other words, the number of fatal or injury accidents in earlier years will be lower. This will cause a decrease in the number of accidents as a whole.
2. The quality of lifespan of the drivers and passengers who might have an accident in earlier years and get wounded, become disabled or die will actually increase, on the contrary.
3. There will be less fatal accidents and fewer injuries.
4. Decrease in loss of younger drivers and passengers will prevent the psychological break down in the relatives of those.
5. Having an accident is also very damaging to children. Even though it is not a 7. The state of death or disabilities of the youngsters will be less.
6. The state of death or disabilities of the youngsters will be less.
7. The relationships with courts and insurance companies as a result of accidents and the materialistic and non-materialistic difficulties of such relationships will also decrease or disappear with the decrease in accidents.
8. The materialistic damages resulting from accidents and their effects on the economy will decrease or totally disappear.
9. Hospital and medication expenses of the casualties will decrease or totally disappear due to the decrease in the number of accidents.
10. The disabilities due to accidents and their caring costs and work productivity loss will decrease or disappear completely.

All the data collected has been interpreted and presented with the aim of clearing the studies to decrease traffic accidents, forming a safer traffic environment and determining the deficiencies in the reasons that cause accidents. The importance of the feasible models that requires less time and material sources will be understood more while preparing action plans on increasing the nonoccurrence duration of accidents. However, some factors such as traffic density, the quality of driving courses, driver’s drug/alcohol addiction, fatigue, and education level will also influence the results if the study is done in different cities at different times and the results are analyzed differently according to these differences. There will be a minimum amount of accidents all the time even though the efforts to prevent them are so intense. Therefore, studies on this vital issue will continue in the future.

### Strengths and limitations of this study

**Strengths**
- All fatal and injury road traffic collision cases of Kayseri region during 5-yr period were included in the study.
- Five-year time period is generally accepted to be sufficient for the evaluation.
- This study was designed to explore the risk factors that influence occurrence time of fatal and injury outcome.

**Limitations**
- This study was only examined risk factors on traffic accident record reports on Kayseri region.
- This study was not designed to explore the risk factors leading to lethal outcome.

## Conclusion

The effects of the determined factors on occurrence duration/time of the accidents were emphasized. Delaying this occurrence time will end up drivers’ involving in accidents much later. Therefore, experience of drivers and their safety will increase as the state of no accidents is delayed.

It was an important factor to determine the nonoccurrence of accidents between the time the license was taken and accidents happen. As determining occurrence time of accidents and lengthening this time means drivers’ having an accident much later. Therefore, experience of the drivers will increase and the safety of all in traffic will be enabled. This also will contribute to the decrease in accidents in the long run.

These data could be useful for determining of risk factors that influence occurrence time of traffic accidents in development of road safety policy framework in Kayseri.

## Ethical considerations

Ethical issues (Including plagiarism, informed consent, misconduct, data fabrication and/or falsification, double publication and/or submission, redundancy, etc.) have been completely observed by the authors.
